# CdS/CdTe Heterostructures for Applications in Ultra-Thin Solar Cells

**DOI:** 10.3390/ma11101788

**Published:** 2018-09-20

**Authors:** Karla Gutierrez Z-B, Patricia G. Zayas-Bazán, Osvaldo de Melo, Francisco de Moure-Flores, José A. Andraca-Adame, Luis. A. Moreno-Ruiz, Hugo Martínez-Gutiérrez, Salvador Gallardo, Jorge Sastré-Hernández, Gerardo Contreras-Puente

**Affiliations:** 1 Escuela Superior de Física y Matemáticas, Instituto Politécnico Nacional, Ciudad de México C.P. 07738, Mexico; karlazb@gmail.com (K.G.Z-B); patriciagzb2006@gmail.com (P.G.Z.-B.); sastrehj@hotmail.com (J.S.-H.); 2 Facultad de Física, Universidad de La Habana, Colina Universitaria, La Habana C.P. 10400, Cuba; odemelo@gmail.com; 3 Departamento de Materiales de Baja Dimensionalidad, Instituto de Investigaciones en Materiales, Universidad Nacional Autónoma de México, Ciudad de México C.P. 04510, Mexico; 4 Facultad de Química, Materiales-Energía, Universidad Autónoma de Querétaro, Querétaro C.P. 76010, Mexico; fcomoure@hotmail.com; 5 Centro de Nanociencias y Micro y Nanotecnologías del IPN, Ciudad de México C.P. 07738, Mexico; andraca1@yahoo.com.mx (J.A.A.-A.); lmorenor@ipn.mx (L.A.M.-R.); humartinez@ipn.mx (H.M.-G.); 6 Departamento Física, Cinvestav-IPN, Ciudad de México C.P. 07360, Mexico; sgh1977@hotmail.com

**Keywords:** sputtering, semiconductors, thin films, optical materials and properties, solar energy materials

## Abstract

The preparation of ultra-thin semi-transparent solar cells with potential applications in windows or transparent roofs entails several challenges due to the very small thickness of the layers involved. In particular, problems related to undesired inter-diffusion or inhomogeneities originated by incomplete coverage of the growing surfaces must be prevented. In this paper, undoped SnO_2_, CdS, and CdTe thin films with thickness suitable for use in ultra-thin solar cells were deposited with a radiofrequency (RF) magnetron sputtering technique onto conductive glass. Preparation conditions were found for depositing the individual layers with good surface coverage, absence of pin holes and with a relatively small growth rate adapted for the control of very small thickness. After a careful growth calibration procedure, heterostructured solar cells devices were fabricated. The influence of an additional undoped SnO_2_ buffer layer deposited between the conductive glass and the CdS window was studied. The incorporation of this layer led to an enhancement of both short circuit current and open circuit voltage (by 19 and 32%, respectively) without appreciable changes of other parameters. After the analysis of the cell parameters extracted from the current-voltage (I-V) curves, possible origins of these effects were found to be: Passivation effects of the SnO_2_/CdS interface, blocking of impurities diffusion or improvement of the band alignment.

## 1. Introduction

With an expected efficiency above 20% [1], conventional, highly absorbing, and non-transparent CdS/CdTe thin film solar cells are among the most promising photovoltaic devices for terrestrial purposes. A typical solar cell uses a 10 µm CdTe absorber layer which has a nearly optimal band gap and a high absorption coefficient [2]; a thin CdS film serves as window and n-type partner. Although the processing of these two materials can be carried out with some different physical or chemical methods [3,4], the sputtering technique has unique advantages, as for example, allowing sequential depositions without vacuum breaking, good control of small growth rates (particularly useful for growing ultra-thin layers) and no wastes generation.

The use of partially transparent CdS/CdTe ultra-thin (UT) solar cells has been the focus of recent research efforts due to the feasibility of their application in different kinds of windows or roofs [5]. For these applications, UT cells should entail: (i) Efficiencies as high as possible (taking into account that a significant part of the radiation is obviously not absorbed in the cell); (ii) aesthetics, as to be compatible with building, sunroofs, etc.; (iii) adequate optical properties in the visible region as demanded by standard regulations tinted windows; and (iv) comfortable indoor buildings environment. These cells are fabricated with the absorber layer whose thickness is thinner than the length of optical absorption and carrier diffusion leading to the desired partial transparency and enhanced carrier collection.

The growth of heterostructures composed by very thin films entails challenges mainly related to thickness uniformity and uncompleted surface coverage. In this paper, RF sputtering was used for the growth of ultra-thin SnO_2_, CdS, and CdTe films with thickness optimized for the requirements of UT solar cell. Heterostructures combining these materials were prepared as well. To illustrate the application of these structures, solar cell devices were fabricated onto conductive glass substrates by adding Au/Cu back contacts. Since the insertion of buffer layers has proven to increase the efficiency and the reproducibility of thick solar cells [6], we studied the influence of an undoped SnO_2_ (u-SnO_2_) buffer layer in the performance of our UT cells. u-SnO_2_ layers have been previously prepared by different techniques such as chemical reactive evaporation [7], chemical vapor deposition [8] or sputtering [9] as per our knowledge, there are no reports on the use of these buffer layers in UT solar cells. Possible origins of the observed influence of the buffer layer in the photovoltaic performance of the devices are explored in this work by the analysis of the cell parameters extracted from the current-voltage curves.

## 2. Materials and Methods 

CdS, u-SnO_2_, and CdTe UT films were deposited with a sputtering system (model V3, Intercovamex, Mexico City, Mexico) equipped with three magnetrons and corresponding 3-in diameter targets (purity of 99.99%). Conductive soda-lime glasses (SLG)/SnO_2_:F (2 nm) (in what follows, TEC15, according to the manufacturer nomenclature, Pilkington, St Helens, United Kingdom) were used as substrates. Before depositing the film, the substrates were ultrasonically cleaned with a warm neutral detergent solution, rinsed with deionized water and isopropyl alcohol, and dried with nitrogen. Deposition was carried out in an Ar atmosphere while maintaining a fixed substrate temperature and RF-power of 250 °C and 80 W, respectively. According to previous growth rate calibration (see Figure S1 in the Supplementary Material), deposition times were 50 min for u-SnO_2_ and CdS and 4 h for CdTe. With these deposition times, the thicknesses of 35, 70 and 650 nm were obtained for u-SnO_2_, CdS, and CdTe, respectively. The thickness of the CdTe layer was selected to balance acceptable optical transmittance (as required for UT solar cells), good homogeneity, and complete surface coverage. Using the growth parameter determined for the individual layers, two different heterostructures were prepared: TEC15/u-SnO_2_/CdS/CdTe and TEC15/CdS/CdTe. With the aim of illustrating the application of these heterostructures, solar cell devices were fabricated with both structures by adding a Cu/Au back contact evaporated onto the CdTe surface.

Optical transmission spectra were measured with a Lambda 35 ultraviolet-visible spectrophotometer (Perkin-Elmer, Mexico City, Mexico) in the range from 330 to 1100 nm. X-Ray diffractograms were taken with a X’Pert PRO PANalytical diffractometer (Almelo, Netherlands) in fixed grazing incidence angle at room temperature and using Cu-Kα radiation (λ = 1.54056 Å). Raman spectra were measured with a Labram HR800 (Horiba Jobin Yvon, Kyoto, Japan) equipment, with an excitation line of 532 nm. Scanning electron microscopy (SEM) images and energy dispersed spectroscopy (EDS) were obtained with a JSM 7800F JEOL system (Kyoto, Japan). For cross-sectional imaging, samples were prepared by polishing and plasma erosion of the broken edge of cut samples. The thicknesses of the films were measured using a profilometer Ambios XP-100 (California, United States). Compositional profiles were obtained on a “time of flight” secondary ion mass spectrometer (SIMS, ION TOF, New York, NY, United States); a 150 μA Cs primary beam with energy of 2 KeV; an area of 400 μm^2^ was used. The room temperature I-V curves were obtained with an Oriel solar simulator Newport model 91160 whose illumination area is 25 cm^2^ using an AM1.5G filter and a radiation intensity of 100 mW/cm^2^.

## 3. Results and Discussion

### 3.1. Individual Layers

**Figure 1:**
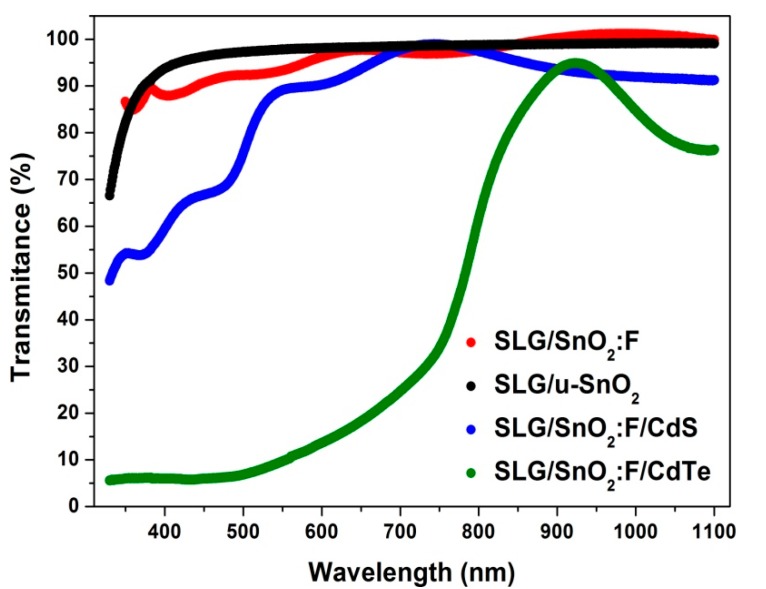
Transmission spectra for the different layers studied in this paper.

With the final objective of inserting them in the heterostructures, we proceeded to characterize the individual layers in terms of their morphology, crystalline structure, and chemical composition. Figure 1 shows the transmittance spectra of the TEC15 substrate, u-SnO_2_, CdS, and CdTe films in the range from 330 to 1100 nm (the sputtered films were measured with the TEC15 substrate as the baseline). As can be observed, u-SnO_2_ and CdS films presented an average optical transmission of 96% and 84%, respectively, when evaluated in a transmission range of 400 to 700 nm. The CdTe layer had an average transmittance of 15%, which is expected to dominate the entire heterostructure device composed of the different films. This value is comparable the one typically reported in the UT solar cells industry. (For example, Sun Well Solar Company sells semi-transparent PV modules with a transmittance of 13.5% in the visible range [10]). No appreciable modifications were observed in the transmission spectra (see supplementary material) from the full heterostructures with and without the buffer layer. This was expected due to the transparency of the u-SnO_2_ layer (see Figure S2 in the Supplementary Material).

**Figure 2:**
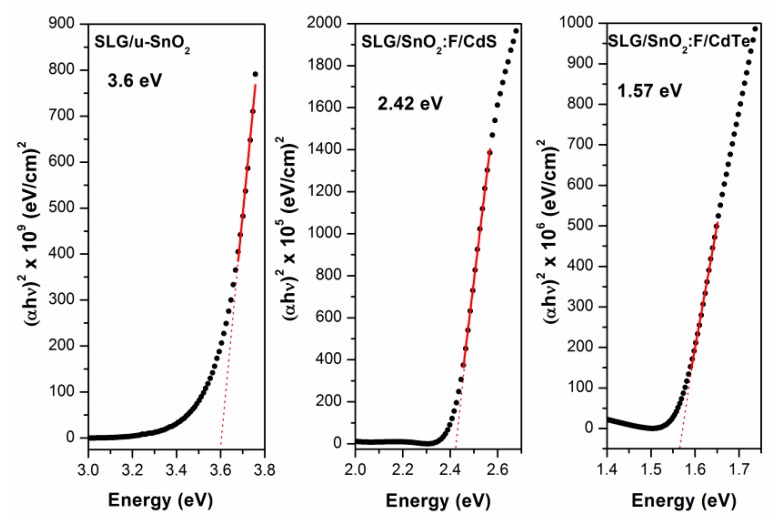
Tauc plots used for band gap calculation of the different materials forming the heterostructure. The extrapolation of the (αhυ)^2^ vs. E linear region fitting is also shown.

The band gaps of the deposited materials were calculated using the low transmission region of the UV-VIS spectra, where a clear absorption edge is noted (Figure 2). $\left(\alpha h\nu \right){}^{n}\;\mathrm{vs}.\;h\nu$ Tauc plots, where α and hν represent the absoption coefficient and photon energy, respectively, with n=2 (as corresponds with direct allowed transitions) were used for these calculations. The band gap values obtained for u-SnO_2_, CdS, and CdTe were 3.6, 2.4 and 1.57 eV, respectively, which are close to those reported in the literature [11,12,13].

**Figure 3:**
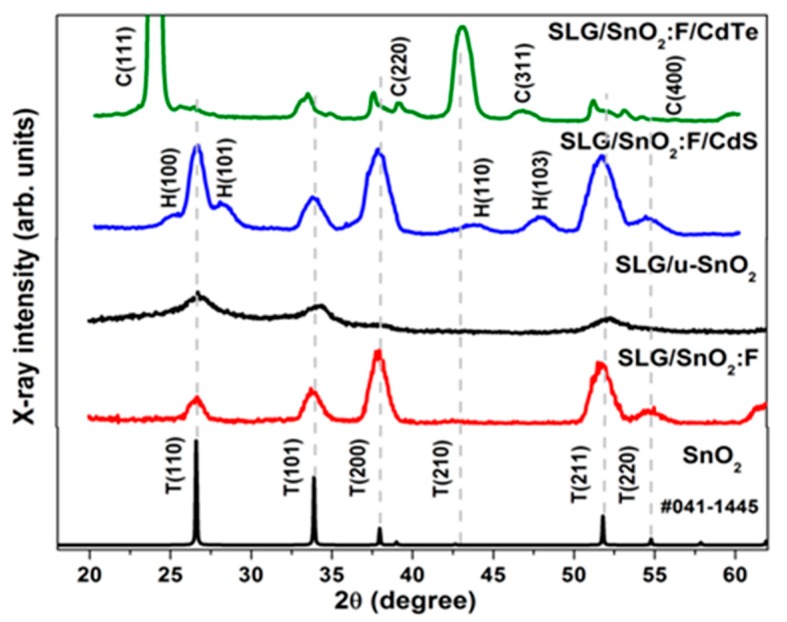
XRD pattern for SnO_2_, CdS, and CdTe deposited onto TEC15. T, C and H stand for tetragonal, cubic, and hexagonal phases.

Diffractograms for the different samples are shown in Figure 3. For SnO_2_, only peaks corresponding to the tetragonal phase (identified as T in the diffractogram) are observed. In the case of CdS, all peaks correspond to the hexagonal phase (PDF 00-041-1049) and are identified as H. On the other hand, CdTe film reveals the presence of a cubic phase (PDF 00-015-0770) (marked as C in the diffractogram). All other peaks in the CdS and CdTe diffractograms, highlighted with dashed lines, correspond to the SnO_2_:F/soda-lime glass substrate as indicated.

**Figure 4:**
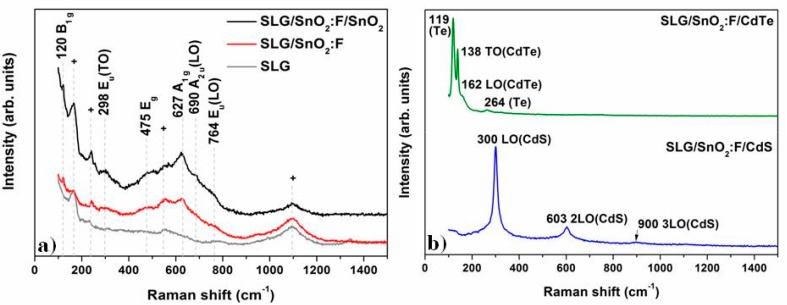
Raman spectra for the samples; (**a**) SnO_2_ sample and the substrate and (**b**) CdS and CdTe samples.

Raman spectra of the u-SnO_2_ film and substrate are shown in Figure 4a. Features at 120, 298, 475, 627, 690 and 764 cm^−1^, corresponding to characteristic SnO_2_ Raman phonons, are observed [14]. Other peaks originating in the soda-lime glass [15] substrate were denoted by +. 

Raman spectra of CdS and CdTe are shown in Figure 4b. For CdS, the peak at 300 cm^−1^ corresponds with the longitudinal optical (LO) phonon mode, while peaks at 603 and 900 cm^−1^ can be assigned to the harmonics 2LO and 3LO, respectively [16]. In the case of CdTe, the peaks located at 138 and 162 cm^−1^, correspond to the transverse optical (TO) and LO CdTe Γ-phonons modes, respectively. The peaks in 119 and 264 cm^−1^ belongs to an undetermined tellurium phase present in the film [17]. 

**Figure 5:**
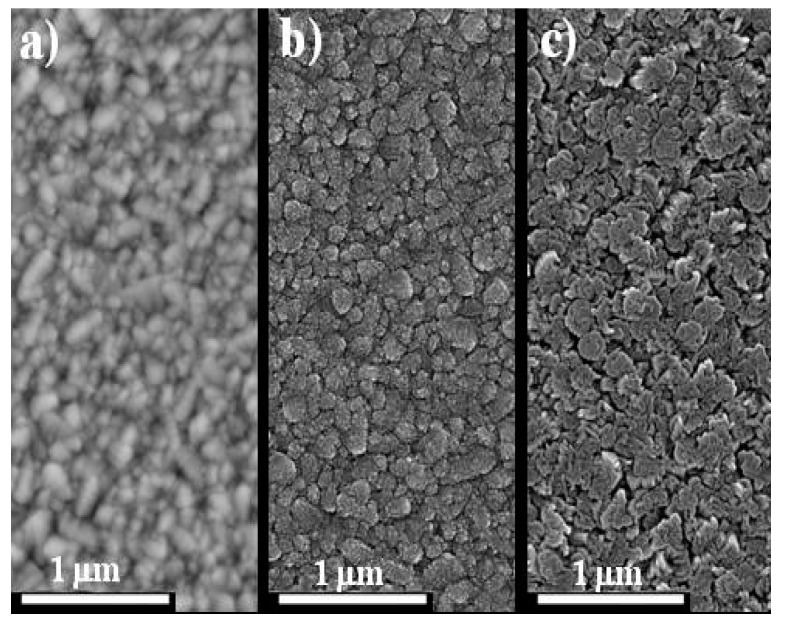
Top view SEM images of the individual layers. (**a**) u-SnO_2_; (**b**) CdS; and (**c**) CdTe films.

Figure 5 shows representative top view SEM images of u-SnO_2_, CdS, and CdTe films. It is remarkable the good surface coverage and the absence of pinholes (as far as the magnification and focus of the images allow) despite the small thickness of the films. In-plane average grain sizes measured from this SEM images were 160, 158 and 177 nm for u-SnO_2_, CdS, and CdTe, respectively (see Figure S3 in the Supplementary Material).

### 3.2. Heterostructures

**Figure 6:**
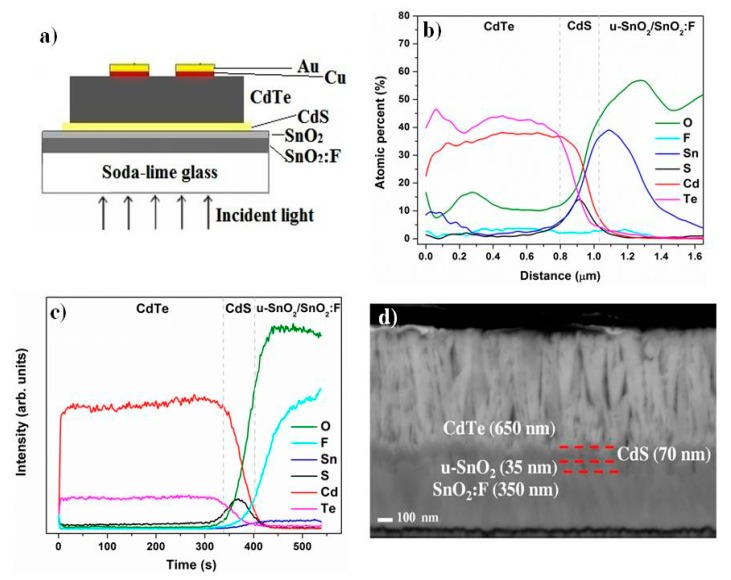
(**a**) The diagram of the solar cell with the buffer layer. Composition profiles of the heterostructures; (**b**) EDS linear map and (**c**) SIMS; (**d**) Cross-section SEM image.

With the same growth parameters used for the individual layers, the heterostructures were fabricated. The schematic representation of the structure is shown in Figure 6a. Composition profiles for a typical heterostructure, determined with EDS linear mapping along the cross-section of the samples and SIMS are shown in Figure 6b,c, respectively. The obtained profiles correspond well with the cross-section SEM micrograph shown in Figure 6d, where relatively abrupt interfaces can be noted. A columnar growth is evident in the CdTe layer. 

To illustrate the application of the heterostructures, a solar cell was fabricated in the superstrate configuration by depositing Cu/Au back contacts unto the CdTe surface as indicated in Figure 6a. (The solar cell without buffer layer was very similar; except for the u-SnO_2_ layer).

**Figure 7:**
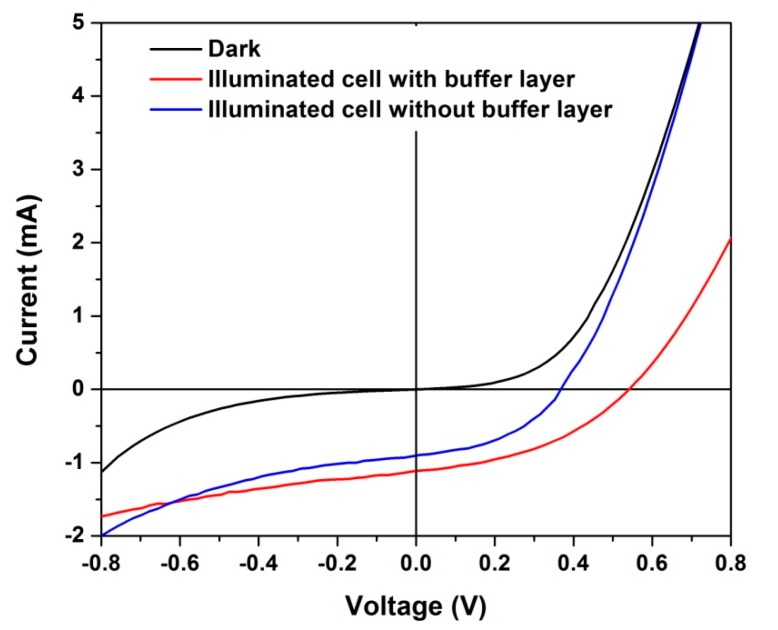
Dark and illuminated I-V curves of the heterostructures.

Dark and illuminated I-V curves of the two heterostructures (with and without u-SnO_2_ layer) are displayed in Figure 7. The device parameters of the solar cell extracted from these curves are summarized in Table 1. Although the device characteristics were not optimized, a clear photovoltaic (PV) effect is observed. The cell with the added u-SnO_2_ layer presents a better PV performance. While fill factor (**FF**) values were practically the same for both devices, the short circuit current (**Isc**) and open circuit voltage (**Voc**) increased by 19 and 32%, respectively, for the cell with the added u-SnO_2_ layer. This presents an efficiency of **η** = 3.75%, 1.7 times higher than that of cell without the buffer layer. As expected, the series resistance of the cell increases with the incorporation of the buffer layer while, remarkably, the shunt resistance is practically not modified. This means that the effect of the buffer incorporation is not related to the elimination of micro short-circuits or leakage currents which should be present in this type of UT cell. Rather, the buffer layer impacts the Voc and Isc as noticed above. The influence of the front contact on the Voc for CdTe cells has been empirically suggested [18] but the reason of this influence is not completely clear. It is speculated that the contribution of the u-SnO_2_ buffer layer is related to the passivation of the SnO_2_/CdS interface and the consequent reduction of the recombination in this region. This should favor the increase in both Isc and Voc as was observed. It cannot be excluded that the u-SnO_2_ buffer also promotes a better band alignment in the structure or helps block impurities diffusion from the TEC-15 substrate, as it has been reported in the literature [19,20].

## 4. Conclusions

Individual layers of undoped SnO_2_, CdS, and CdTe with thickness and optical transmission adapted to operate in UT solar cells were obtained by RF sputtering. These layers were characterized and successfully incorporated into heterostructures with relatively abrupt interfaces and good surface coverage. Solar cell devices were fabricated with these heterostructures which shown reasonable photovoltaic parameters, especially if it is taking into account that many device related parameters were not properly optimized. For example, thermal treatment in CdCl_2_ or contact annealing should be further optimized with the aim of obtaining more efficient devices. In this work, it was clearly observed that the inclusion of an undoped SnO_2_ buffer layer led to an appreciable increase of the open circuit voltage and a moderate increase in the Isc. The increase in series resistance caused by the introduction of the additional layer is more than compensated by the Voc and Isc increases. Overall, the efficiency of the cell improved from 2.1% to 3.7%. These changes promoted by the buffer layer are probably due to passivation effects of the SnO_2_/CdS interface, blocking of diffusion impurities from the substrate or to improvement of the band alignment. 

## Figures and Tables

**Table 1:** Extracted PV parameters from the I-V curves of the CdS/CdTe devices.

Samples	Voc (mV)	Jsc (mA/cm^2^)	FF (%)	η (%)	Series Resistance (Ω cm^2^)	Shunt Resistance (Ω cm^2^)
Without buffer layer	369	11.3	43.2	2.1	2.9	97.8
With buffer layer	542	13.9	41.1	3.7	5.4	95.7

## Supplementary Materials

The following are available online at http://www.mdpi.com/1996-1944/11/10/1788/s1, Figure S1: Growth rate determined for each material. (a) u-SnO_2_ and CdS, and (b) CdTe; Figure S2: Transmission spectra for the CdTe layer and the heterostructures with and without buffer layer studied in this paper; Figure S3: Histograms of the grain size values in each layer.
