# Supplementary material for: CdS/CdTe Heterostructures for Applications in Ultra-Thin Solar Cells
Source: Materials (Basel). 2018 Sep 20;11(10):1788. doi: 10.3390/ma11101788 (PMC6213385; doi:10.3390/ma11101788)
Supplement: Supplementary file 1 [file materials-11-01788-s001.pdf]

Supplementary material

Growth rate determined for each material. Figure S1a u-SnO_2 and CdS and Figure S1b CdTe. All the experiments were carried out in Ar atmosphere; the substrate temperature and RF-power were fixed at 250 °C and 80 W, respectively.

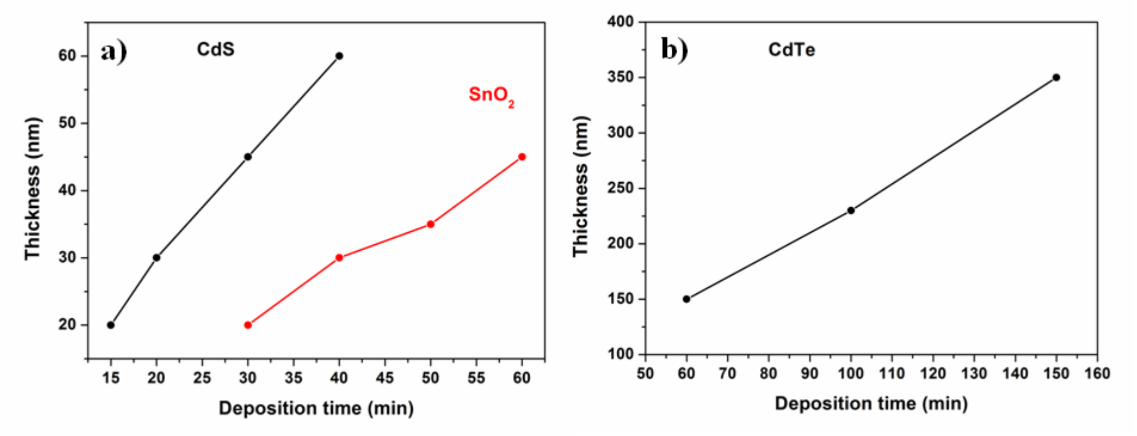

Figure S1. Growth rate determined for each material. (**a**) u-SnO_2 and CdS and (**b**) CdTe.

The transmission spectra were not appreciably modified by the insertion of the buffer layer. This was expected given the transparence of the u-SnO_2 layer. Figure S2 shows the transmission spectra for the CdTe layer and the heterostructures with and without buffer layer studied in this paper.

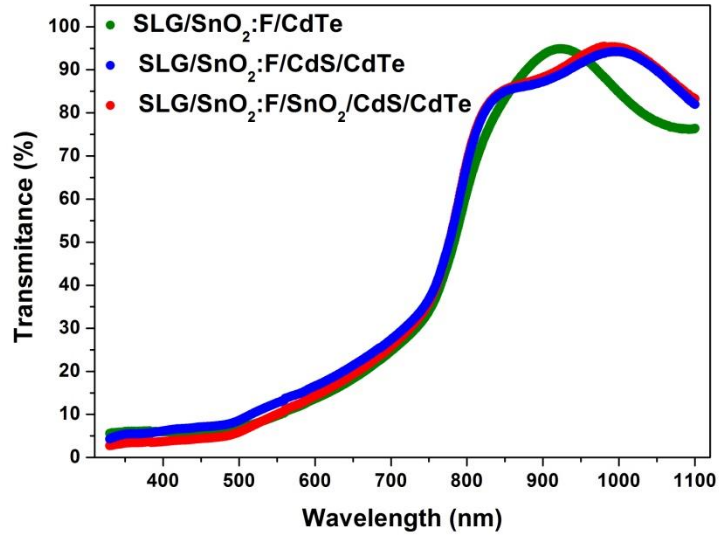

Figure S2. Transmission spectra for the CdTe layer and the heterostructures with and without buffer layer studied in this paper.

Figure S3 shows the statistics on the in-plain grain sizes in each layer. A total of 200 grains were measured and a histogram was created from the data. The data were adjusted with a Gaussian shape.

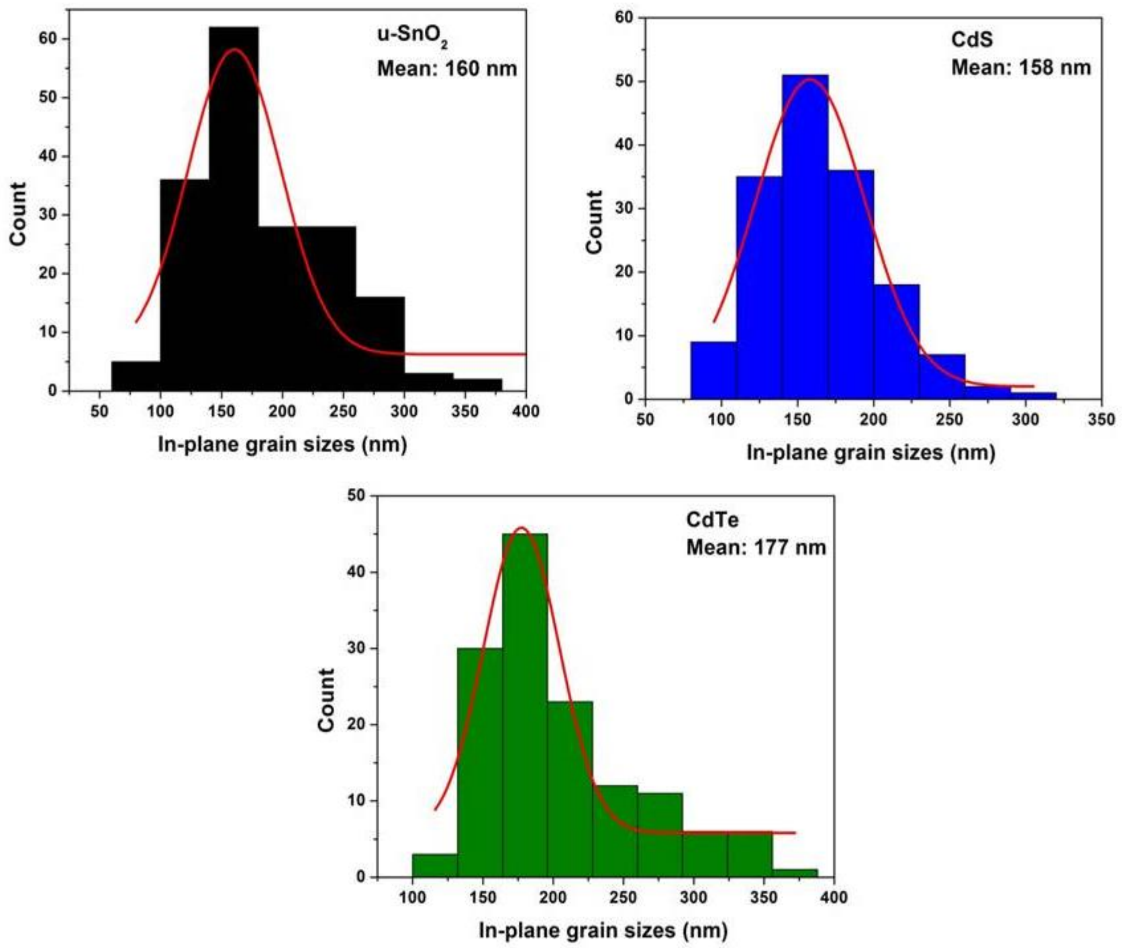

Figure S3. Histograms of the grain size values in each layer.
